# Identification of process relevant miRNA in CHO cell lines - Process profiling reveals interesting targets for cell line engineering

**DOI:** 10.1186/1753-6561-7-S6-P81

**Published:** 2013-12-04

**Authors:** Fabian Stiefel, Matthias Hackl, Johannes Grilliari, Friedemann Hesse

**Affiliations:** 1 Institute of Applied Biotechnology Biberach, Germany; 2 University of Natural Resources and Life Sciences, Institute for Applied Microbiology, Vienna, Austria

## Introduction

MicroRNAs (miRNAs) are small RNAs which function as regulators of posttranscriptional gene expression by binding to their mRNA targets [1]. MiRNAs are involved in crucial regulations of many signaling and metabolic pathways. In difference to other interfering RNAs (RNAi), miRNAs can target many mRNA, thus having an increased impact on regulation of gene expression. These properties of miRNAs makes them interesting and promising targets for biomarkers and cell line engineering [2,3]. Therefore, we studied miRNA profiles during different culture phases and process conditions and investigated the potential of differentially expressed miRNAs as targets for process optimization. This may help to pave the way to introduce a new layer of control for cell line engineering.

## Results

For miRNA target selection a strain from Chinese hamster ovary cells (CHO-DG44) was cultivated in a 2L bioreactor (Biostat B plus, Sartorius Stedim, Germany) in Batch mode and two different process conditions, control runs and temperature shift. For the control runs temperature was maintained at 37°C all time, while for the temperature shift the temperature was reduced to 30°C. Isolated RNA was analyzed using microarray technology (PowerScanner and HS 400 Pro Hybridisation station, Tecan, Germany and a cross-species chip containing miRNAs from human, mouse and rat, University of Graz) and the best differential expressed miRNA were cross-validated with qRT-PCR.

**Figure 1:**
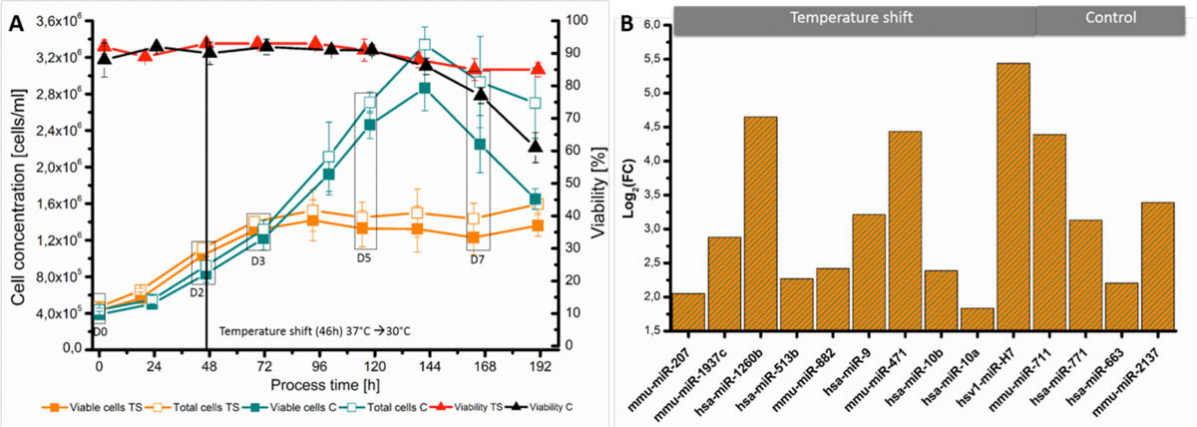
**A) Sample creation with final protocol of CHO-DG44 cells in 2L bioreactors**. **B) **Summary of miRNA targets of microarray analysis for validation with qRT-PCR

The optimized bioreactor protocol, shown in Figure 1 A, each process condition included two independent biological replicates. The control runs show good growth behavior to a maximal viable cell concentration of 2.9 × 10^6 ^cells/ml. Reducing temperature from 37°C to 30 °C resulted in a clear inhibition of cell growth by sustained viability. From each time point of the different culture phases a sample was taken and total RNA was purified.

Purified samples were labeled and then analyzed on a cross species miRNA microarray chip. Differential expression was always calculated between the time points for the respective culture stage compared to day zero (shown in Table 1). The temperature shift from 37°C to 30°C after 46 hours had a high impact on the miRNA profile with 22 differentially expressed miRNAs for the early response from day three (D3-D0). In the late response (D5-D0) only three miRNAs and 18 miRNAs in the very late response (D7-D0) were differentially expressed. Two miRNAs were constantly expressed after shifting the temperature. In the control run at 37°C the number of differentially expressed miRNA was increasing during the course of the cell culture ranging from two miRNAs for the early to medium exponential phase, 12 miRNAs for the late exponential phase to 28 miRNA in the declining phase.

**Table 1:** Summary of differentially expressed miRNAs in the control run and the temperature shift

	Amount of differentially expressed miRNA	
	Control	Temperature shift
D2-D0	0	0
D3-D0	2	22
D5-D0	12	3
D7-D0	28	18

To validate microarray normalization and results, differentially expressed miRNAs from the microarray analysis were cross-validated with qRT-PCR. This validation was conducted for respective miRNA of the time points before and after the temperature shift. Fold changes of mmu-miR-207 (Log_2 _FC of microarray was 2.0 and 2.9 for qRT-PCR) and mmu-471-5p 207 (Log_2 _FC of microarray was 4.4 and 5.0 for qRT-PCR) obtained from microarray and qRT-PCR technology were very comparable and showed same trends. This indicates that the microarray results can be used for a deeper analysis of the differentially expressed miRNAs.

During a batch run, culture parameters are changing. In order to investigate the impact of these changes to miRNA profiles, time course of differential expression of miRNA during the different cell culture phases were analyzed. For the time courses of ten miRNAs in the temperature shift most of the miRNAs showed their highest differential expression shortly after the reduction of the temperature. Some miRNAs keep their level of differential expression, some return to normal levels three days after the temperature shift. One miRNA is differential expressed at the end of the observed culture phase. In the control run the number of differential expressed miRNAs and the fold change of the differential expression is increasing during progressing culture phase.

Figure 1 B shows the differential expression of ten miRNA directly after the temperature shift and four miRNAs for the control runs between day zero and the declining phase at day seven. For the temperature shift differential expression ranges from log_2_FC 1.7 to 5.4 and 2.0 to 4.4 for the control runs. This selection of miRNAs presented here may be interesting candidates for further investigation using miRNA overexpression/inhibition and phenotype studying.

## Conclusion

As a conclusion, with optimized bioreactor protocols it was possible to establish miRNA profiles of CHO-DG44 cells for different culture phases on cross species microarray chips. The number of differential expressed miRNAs was increasing by progressing of the culture phase. Additionally, the impact of a temperature shift on the profiles revealed several highly differentially expressed miRNA. Some of these miRNAs were already cross-validated with qRT-PCR which confirmed the results from the microarray experiment. MiRNA targets of these two experimental approaches will help to increase the knowledge of the role of miRNAs during a bioreactor process and might pave the way for their use in cell line engineering.
